# Dimethyl Isosorbide: An Innovative Bio-Renewable Solvent for Sustainable Chromatographic Applications

**DOI:** 10.3390/molecules30132713

**Published:** 2025-06-24

**Authors:** Aleksandra Damjanoska, Kristina Mitreska, Matilda Petrova, Jelena Acevska, Katerina Brezovska, Natalija Nakov

**Affiliations:** 1 Institute of Applied Chemistry and Pharmaceutical Analysis, Faculty of Pharmacy, Ss Cyril and Methodius University in Skopje, Mother Tereza 47, 1000 Skopje, North Macedonia; kmitreska23@gmail.com (K.M.); jelena_petrusevska@ff.ukim.edu.mk (J.A.); kami@ff.ukim.edu.mk (K.B.); natalijan@ff.ukim.edu.mk (N.N.); 2 Manufacturing Technical Operations, ALKALOID AD, Blvd. Aleksandar Makedonski 12, 1000 Skopje, North Macedonia; mpetrova@alkaloid.com.mk

**Keywords:** dimethyl isosorbide, bio-renewable solvent, liquid chromatography, green chemistry, sustainability, partition coefficient, viscosity, pharmaceutical analysis

## Abstract

The increasing environmental concerns and regulatory restrictions on toxic conventional solvents have driven the search for sustainable alternatives. Dimethyl isosorbide (DMI), a bio-renewable solvent, has shown potential as a replacement for short-chain glycol ethers, although its use as solvent in liquid chromatography (LC) is underexplored. This study presents a physicochemical characterization of DMI with a particular focus on its application as an innovative solvent in LC analyses. The partition coefficient (*log P* = −0.44) was determined using the OECD 107 method, and viscosity measurements for DMI and its mixtures with water and ethanol were conducted at 25 °C, 40 °C, and 60 °C. Viscosity ranged from 1.28 mPa·s at 60 °C to 2.62 mPa·s at 40 °C. The Central Composite Face 2^3^ experimental design for studying the chromatographic behavior of DMI confirmed that 50% (*v*/*v*) DMI can be effectively utilized in the mobile phases, at a column temperature of 40 °C, with backpressures ranging from 160 to 300 bar and a UV cut-off at 240 nm. Its effectiveness as an eluent in LC was demonstrated for the quantification of methylparaben and propylparaben in pharmaceutical formulations. This study highlights DMI’s promise as a sustainable bio-renewable alternative to conventional organic solvents used as eluents in LC, supporting eco-friendly practices in pharmaceutical analysis.

## 1. Introduction

As environmental concerns surrounding conventional organic solvents grow, there is a significant shift towards more sustainable, bio-renewable alternatives [1]. Conventional solvents like acetonitrile (ACN) and methanol (MeOH) are commonly used in liquid chromatography (LC), but their toxicity, flammability, and environmental impact pose long-term threats to human health and ecosystems. Their widespread use contributes to pollution and leads to high energy consumption in solvent disposal and recovery [1,2]. With increasing pressure for greener, more energy-efficient chemical processes, research into environmentally friendly solvents has surged over the last three decades [3,4].

Green chemistry has become a cornerstone of modern science and industry, aiming to reduce harmful environmental impact and improve the safety of both industrial practices and the chemicals involved. A critical aspect of this effort is green analytical chemistry (GAC), which refines analytical techniques to ensure they are non-toxic, efficient, and sustainable [5]. As regulatory standards tighten, industries are increasingly motivated to adopt eco-friendly practices that also offer competitive market advantages. GAC promotes the development of analytical methods that align with global sustainability goals [6,7].

In LC analyses, organic solvents such as ACN and MeOH are favored for their physicochemical properties, including excellent UV transmission, low viscosity in aqueous solutions, and high solubility in both polar and non-polar phases [2]. However, these solvents have significant drawbacks: ACN is volatile, toxic, and flammable, while MeOH, though more biodegradable, remains hazardous due to its toxicity and problematic disposal [2]. This has driven interest in bio-renewable solvents derived from biomass, such as agricultural residues, forestry by-products, seaweed, and food waste, as safer and more sustainable alternatives [8,9,10].

The CHEM21 selection guide is an established guideline for the evaluation of classical and less classical solvents, such as bio-renewable solvents [11,12]. According to this guideline, the environmental, health, and safety properties of solvents are scored in several categories including VOC (volatile organic compounds) emissions, flammability, toxicity, and biodegradability. These scores reflect the solvent’s sustainability and overall impact, with a scale ranging from one to four, where one represents a less sustainable or more hazardous solvent, and four indicates a more sustainable or less hazardous one. The individual scores are then expanded to a 1–10 scale to provide a finer classification [11,12]. The CHEM21 methodology also incorporates composite scores to categorize solvents into color-coded categories (green, yellow, red), making it easier to identify which solvents are most suitable for green chemistry applications. The comprehensive CHEM21 categorization system ensures a detailed assessment of the sustainability and safety profile of solvents, allowing for informed decision-making regarding its use in various applications, including LC [11,12].

Nowadays, researchers are increasingly turning to bio-renewable solvents for use as eluents in LC, as part of the broader trend toward greener and more sustainable analytical methods [10,13]. Recent studies have demonstrated the potential of the bio-based solvent Cyrene as a co-solvent with ethanol (EtOH) in reverse-phase high-performance LC (RP-HPLC). However, Cyrene’s higher viscosity compared to traditional solvents such as ACN and MeOH poses a challenge, as it may increase backpressure in chromatographic systems [14]. In addition to Cyrene, dimethyl carbonate (DMC) has also emerged as a promising green solvent. DMC has been successfully applied as the sole organic eluent in SPE and RP-UPLC for the simultaneous determination of theobromine and caffeine in tea extracts, offering high recoveries, good precision, and significantly improved environmental metrics [15]. Beyond this, recent studies have further demonstrated the versatility and sustainability of DMC across a range of chromatographic techniques. For instance, DMC has shown excellent compatibility with inductively coupled plasma mass spectrometry (ICP-MS), where conventional organic solvents often compromise sensitivity or require system modification. At only 10% (*v*/*v*) concentration, DMC enabled stable plasma conditions and markedly enhanced elution strength, reducing retention times up to 40-fold compared to MeOH, while maintaining instrument cleanliness and sensitivity over prolonged use [16].

Moreover, DMC has been explored as a non-toxic, aprotic modifier in both hydrophilic interaction chromatography (HILIC) and normal-phase LC. It provided superior retention behavior compared to ethyl acetate and enabled enhanced UV detection of phthalates. In HILIC mode, DMC offered improved plate counts and peak symmetry over ACN, along with the ability to resolve complex mixtures such as positional isomers of hydroxybenzoic acids and food preservatives [17].

Importantly, DMC’s applicability extends to preparative-scale liquid chromatography, particularly in the purification of therapeutic peptides. Compared to ACN, EtOH, and isopropanol, DMC delivered the best productivity at high purities, due to its higher elution strength and lower toxicity. This positions DMC as a viable, sustainable alternative to traditional solvents not only for analytical but also for industrial-scale chromatographic processes [18,19]. These findings collectively support the growing role of bio-renewable and environmentally benign solvents in reshaping sustainable chromatographic practices across diverse analytical and preparative contexts.

Alongside Cyrene and DMC, dimethyl isosorbide (DMI), a bio-renewable solvent synthesized from renewable resources like D-sorbitol (a sugar alcohol derived from glucose), offers excellent solubility, moderate viscosity, and negligible bioaccumulation, making it an attractive green alternative for industrial processes such as polymer membrane preparation and organic synthesis [20,21]. DMI has shown potential as a co-solvent, enhancing the solubility of nonpolar compounds [22,23]. While its use in pharmaceuticals and cosmetics is well-documented, its potential as an eluent in chromatographic analyses remains largely unexplored, despite its favorable physicochemical properties [23].

The aim of this study was to investigate the potential application of the bio-renewable solvent DMI as an eluent in chromatographic analyses. This research was focused on characterizing the key physicochemical properties of DMI using the CHEM21 methodology and evaluating its impact on critical chromatographic parameters. To determine the relationship between viscosity and chromatographic parameters, a Design of Experiments (DoE) approach was employed. Additionally, the feasibility of using DMI as an eluent in sustainable chromatographic methods for determining the content of preservatives in three different oral liquid formulations was tested. Through this research, we aim to demonstrate that DMI offers a sustainable and environmentally friendly alternative to conventional solvents, advancing the transition to sustainable practices in analytical chemistry.

## 2. Results and Discussion

### 2.1. Evaluation of DMI According to CHEM21 and SDS Lists

From an environmental and safety standpoint, DMI exhibits a favorable profile compared to conventional solvents such as MeOH and EtOH (Table 1), particularly in applications where sustainability and operator safety are priorities [11,12].

One of DMI’s most prominent advantages lies in its very low VOC emissions (rated ten), significantly outperforming MeOH (rated three) and EtOH (rated four). This makes DMI especially attractive for use in low-emission and environmentally controlled laboratory settings, in line with the principles of green chemistry [11,12]. In terms of aquatic impact, DMI (rated nine) performs comparably with EtOH (rated nine) and only slightly lower than MeOH (rated ten), indicating low environmental toxicity in aqueous environments [12]. However, its air impact (rated six) is moderate compared to MeOH (seven) and EtOH (five), positioning DMI as a middle-ground choice for air quality considerations [12].

With regard to health hazard, although DMI is not classified as hazardous under Regulation (EC) No 1272/2008, exposure may still cause irritation to the eyes and skin, and inhalation of aerosols should be avoided [24]. Importantly, DMI does not contain components considered persistent, bio accumulative, or endocrine-disrupting. Its health hazard score (rated four) is the same as that of MeOH, yet DMI is markedly less toxic—unlike MeOH, which is known to cause serious systemic effects such as visual impairment, CNS depression, or death in cases of significant exposure [25]. Ethanol, by contrast, carries a health hazard rating of ten, reflecting a generally low risk in standard laboratory usage. Nevertheless, its flammability and potential for misuse remain concerns [12].

DMI’s strengths become even more pronounced in categories related to occupational safety: it boasts a low exposure potential (rated nine), indicating minimal risk under typical laboratory conditions, compared to MeOH (scored six) and EtOH (scored eight) [11]. It also demonstrates very low flammability and explosion risk (rated nine), which is superior to MeOH (scored five) and EtOH (scored six), enhancing its suitability for use in high-safety analytical environments. Though DMI’s chemical stability (reactivity rated eight) is slightly below the ideal values seen with MeOH and EtOH (both rated ten), it remains adequately stable for use in chromatographic and laboratory procedures [12].

In terms of end-of-life management and sustainability, DMI presents a moderate biotreatability score (rated five), outperforming both MeOH and EtOH (rated three), which supports its inclusion among green solvents from a wastewater treatment perspective. However, it shows limited recycling potential (rated four), compared to EtOH (five), and a low incineration suitability (rated four), similar to MeOH and lower than EtOH (5) [12]. Additionally, ready biodegradability tests following OECD TG 301 F indicate that DMI is not readily biodegradable, showing 0% degradation over 28 days under standard conditions [26]. 

However, DMI does not surpass MeOH and EtOH across all performance indicators, but it clearly stands out in key areas such as low VOC emissions, operator safety, low flammability, and moderate environmental persistence. Its moderate health hazard, when compared to the significantly higher toxicity of MeOH and slightly lower hazard of EtOH, further underscores its balanced and responsible profile for analytical applications in controlled laboratory environments. As such, DMI represents a viable and sustainable alternative to conventional solvents, particularly in scenarios where both environmental impact and user safety are critically evaluated.

Beyond its comparison with conventional solvents such as MeOH and EtOH, DMI was also assessed against other prominent bio-based solvents, including dimethyl carbonate (DMC) and Cyrene, both of which are increasingly recognized for their green chemistry potential. According to the data presented in Table 1 the selection of DMI as the solvent of choice was due to its comprehensive and balanced safety profile. While DMC offers excellent chemical stability and negligible toxicity, DMI integrates a broader range of safety-relevant attributes [12]. These include exceptionally low VOC emissions, minimal flammability and explosion risk, low exposure potential, and favorable environmental compatibility in aquatic systems and wastewater treatment. Unlike approaches that prioritize only low intrinsic toxicity, the selection of DMI reflects a more holistic risk mitigation strategy that considers multiple dimensions of laboratory and occupational safety. Thus, DMI emerges as a more versatile and responsible solvent choice for environmentally conscious and safety-critical analytical applications. Notably, DMI and Cyrene, already explored as an eluent in RP-HPLC, exhibit nearly identical safety and environmental profiles, with only minor differences in terms of reactivity and exposure potential. This further underscores DMI’s suitability as a green alternative in chromatographic applications.

**Figure 1:**
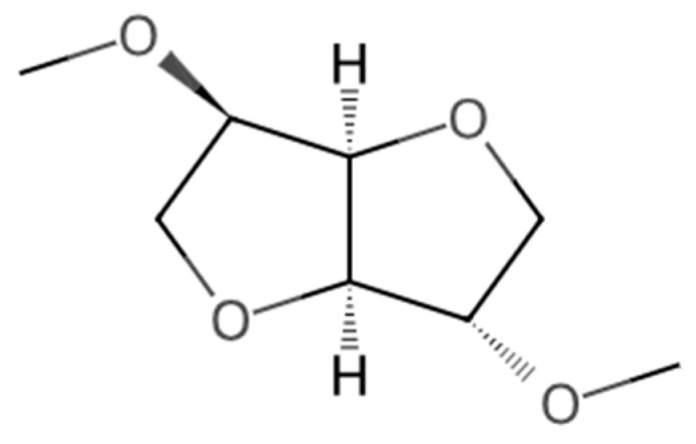
Molecular structure of DMI [27].

According to the data presented in Table 2, several physicochemical properties of DMI (Figure 1) are of particular importance for its application as a solvent in chromatographic methods, especially in HPLC. One key property is viscosity, which significantly affects fluid dynamics within chromatographic columns. Although specific viscosity values for DMI are not reported in the available safety data sheets (SDS), experimental determination of this parameter, across a temperature range from ambient to 60 °C, is essential. Viscosity directly influences the applicable flow rate of the mobile phase and, consequently, the column backpressure (*P*_column_), which must be precisely controlled during method optimization.

**Table 2:** Information on basic physical and chemical properties of DMI, H_2_O, EtOH and MeOH—extracted from SDS lists [24,25,28,29].

Property	DMI	H_2_O	EtOH	MeOH
Appearance	Clear, colorless liquid	Clear, colorless liquid	Clear, colorless liquid	Clear, colorless liquid
Odor	No significant odor	No data available	Characteristic	Characteristic
Odor threshold	No data available	No data available	No data available	No data available
pH	No data available	6.0–8.0 at 25 °C	7.0 at 10 g/L at 20 °C	No data available
Melting point/Freezing point	−84 °C	0.0 °C	−114.0 °C	−97.8 °C
Boiling point	240 °C	100.0 °C	78.29 °C	64.7 °C
Flash point	116 °C	Not applicable	13 °C	9.7 °C
Viscosity	6.62 mPa·s at 25 °C (range: 9.47 at 15 °C to 2.19 at 70 °C) [22]	No data available	1.2 mPa·s at 20 °C	0.544–<0.59 mPa·s at 25 °C
Water solubility	2000 g/L at 20 °C	Completely miscible	1000 g/L at 20 °C	1000 g/L at 20 °C
Vapor pressure	0.073000 mmHg (0.0973 hPa) at 25 °C. (est) [30]	No data available	57.26 hPa at 19.6 °C	169.27 hPa at 25 °C
Vapor density	No data available	No data available	No data available	1.11
Density	1.166 g/cm^3^ at 20 °C	1.000 g/cm^3^ at 3.98 °C	0.79 g/cm^3^ at 20 °C	0.79 g/cm^3^ at 20 °C
Partition coefficient (log P)	−0.423 (est) [30]	−0.467	−0.35	−0.77
Autoignition temperature	285 °C	No data available	363–425 °C	455.0 °C
Decomposition temperature	No data available	No data available	Distillable at normal pressure	Distillable at normal pressure

Another critical parameter is the boiling point of DMI, which is relatively high at 240 °C [24]. While this thermal stability can be beneficial for applications involving elevated temperatures, it represents a major drawback in pharmaceutical and preparative contexts, where solvent removal is often required. The high boiling point implies increased energy consumption for evaporation, thereby reducing the environmental friendliness (“greenness”) and cost-efficiency of the process. In contrast, more commonly used solvents such as water (100 °C), EtOH (78.3 °C), and MeOH (64.7 °C) offer much more efficient evaporation profiles [25,28,29].

DMI exhibits a water solubility of 2000 g/L at 20 °C [24], categorizing it as water-soluble. In contrast to DMI, EtOH and MeOH exhibit lower reported solubility values in water (1000 g/L) [25,28]. The relatively high-water solubility of DMI supports its favorable compatibility with aqueous mobile phases, minimizing the risk of phase separation or precipitation. This characteristic makes DMI particularly suitable for use in mixed solvent systems, especially in RP-HPLC applications where aqueous-organic solvent mixtures are commonly employed.

Although DMI is classified as flammable, with a flash point of 116 °C [24], this value is relatively high compared to EtOH (13 °C) [28] and MeOH (9.7 °C) [25], suggesting that DMI poses lower volatility and flammability risks under standard laboratory conditions, provided that appropriate handling procedures are followed.

Moreover, although reliable predictive estimations exist [30], *log P* of DMI has not yet been experimentally determined. This parameter is essential for predicting solvent-stationary phase interactions, particularly when analyzing hydrophobic or amphiphilic analytes. The absence of such data may limit DMI’s applicability in certain chromatographic systems. Hence, determining the *log P* of DMI could enhance its potential use, especially in the development of complex or targeted separation methods.

### 2.2. Investigation of Miscibility of DMI with Water

The miscibility of DMI with water (H_2_O) was evaluated by preparing mixtures at varying ratios (10:90, 20:80, 30:70, 40:60, and 50:50 *v*/*v* and vice versa) at room temperature. All the the tested mixtures formed homogeneous solutions without any phase separation, indicating that DMI is fully miscible with H_2_O across the entire range of ratios investigated. This behavior can be attributed to the polar nature of both DMI and H_2_O. DMI, being a polar solvent due to its ether and alcohol functional groups, interacts favorably with H_2_O molecules through hydrogen bonding. These interactions likely contribute to the observed miscibility in all tested concentrations. The absence of phase separation suggests that the intermolecular forces between DMI and H_2_O are strong enough to maintain a single, homogeneous phase, even when the proportions of DMI and H_2_O are varied. This finding is important in the context of analytical purposes where a stable, uniform mixture is desired.

### 2.3. Investigation of the Viscosity of DMI and Its Mixtures with H_2_O and EtOH

The viscosity of DMI in different mixtures of H_2_O and EtOH provides key insights into its potential use as an eluent in LC analyses. As expected, viscosity decreases with increasing temperature for all tested samples, with pure DMI showing a significant reduction in viscosity from 6.70 mPa·s to 2.79 mPa·s, as the temperature increases from 25 °C to 60 °C (Table 2). Mixtures containing both H_2_O and DMI (e.g., 70% DMI:30% H_2_O, 50% DMI:50% H_2_O) also exhibit similar reductions in viscosity with temperature (Table 3), which is crucial because lower viscosity facilitates the flow of eluents through chromatographic columns, reducing *P*_column_ and increasing analysis speed. The viscosity of mixtures of DMI and H_2_O is inversely proportional to the H_2_O content. The highest viscosity of 6.38 mPa·s at 25 °C was observed for the mixture containing 70% DMI and 30% H_2_O; while the mixture of 30% DMI and 70% H_2_O provides lowest viscosity value of 2.38 mPa·s at 25 °C. The high viscosity of DMI could result in increased *P*_column_ during HPLC runs, which may be problematic for UPLC applications. This characteristic limits DMI’s use in fast separations and modern column technologies with narrow internal diameters and small particle sizes (below 5 µm). This suggests that lower concentrations of DMI may be more useful for LC methods that require low viscosity to minimize *P*_column_. For comparison, the viscosity of pure water at 25 °C is approximately 0.89 mPa·s, and that of EtOH is about 1.07 mPa∙s at the same temperature [31]. These values highlight that even the most diluted DMI mixture (30% DMI) still exhibits higher viscosity than either H_2_O or EtOH.

This trend suggests that lower concentrations of DMI may be more suitable for LC methods where the minimization of mobile phase viscosity is essential to reduce *P*_column_ and improve efficiency.

By adding EtOH to DMI and H_2_O mixtures, viscosity reduction is observed in most cases (Table 4). The role of EtOH in reducing viscosity is significant as it aids in creating a mobile phase, which is often desired in chromatographic analyses to ensure optimal separation and reduced *P*_column_. Mixtures with lower viscosity (such as 30% DMI:70% H_2_O) are likely more suitable for routine chromatographic analysis because they result in lower *P*_column_, faster elution times, and reduced column wear. However, the higher viscosity of mixtures containing 70% DMI could be beneficial for more complex analyses, where the interaction between the stationary and mobile phases might require a more viscous eluent for better resolution. Considering the relationship between viscosity and flow rate, DMI mixtures at lower concentrations (e.g., 50% DMI:50% H_2_O) or those with added EtOH (e.g., 33.33% DMI:33.33% H_2_O:33.33% EtOH) could offer a good compromise between efficient elution and resolution, especially at moderate temperatures (40–60 °C). These mixtures are likely to be ideal candidates for general use in chromatographic applications, where faster analysis and moderate *P*_column_ are priorities.

The viscosity profile of DMI-based mixtures with H_2_O and EtOH showed that adjusting the ratio of these solvents could significantly impact the chromatographic performance. Depending on specific analytical requirements, the most appropriate mixture can be selected to balance viscosity, *P*_column_ and elution speed.

### 2.4. Determination of the UV Cut-Off of DMI and Its Implications for Chromatographic Use

**Figure 2:**
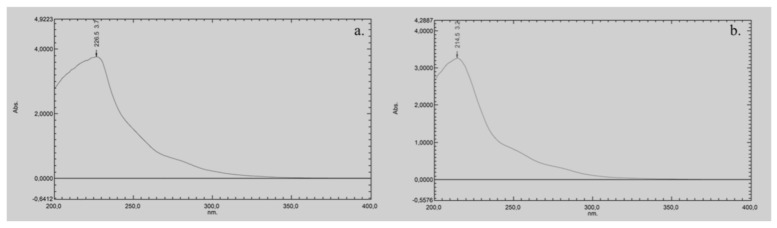
*UV-vis* spectrum of: (**a**) pure DMI, and (**b**) a mixture of DMI and water (50:50 *v*/*v*).

The UV cut-off of pure DMI, defined as the wavelength at which the absorbance reaches 1.0 AU in a 1 cm path length cell, is approximately 260 nm (Figure 2a). For the 50:50 (*v*/*v*) DMI:H_2_O mixture, the UV cut-off is slightly lower, at around 240 nm (Figure 2b). This moderate shift toward shorter wavelengths suggests that the addition of water reduces the overall absorbance of the solvent system, likely due to hydrogen bonding and other intermolecular interactions that alter DMI’s electronic environment.

Comparing the UV cut-off value of DMI with other green solvents (like EtOH, glycerol, Cyrene or DMC) is important because these values directly influence their suitability for UV-based detection in chromatographic applications. EtOH has a UV cut-off of around 210 nm [8,11], making it a widely used green solvent in chromatographic methods. Glycerol has a UV cut-off of around 207 nm [8,32], thus it is suitable for UV detection at lower wavelengths. Cyrene has a high UV cut-off of around 350 nm, which limits its use for UV-based detection, especially at shorter wavelengths [8,12]. Consequently, cyrene is generally employed in mixtures with water or other solvents to lower its UV absorption [12]. DMC has a UV cut-off of approximately 220 nm [16], which places it between EtOH and cyrene in terms of UV transparency.

Although, the UV cut-off of the DMI:H_2_O mixture (240 nm) is significantly higher compared to EtOH, DMC or glycerol, it still allows UV detection above this wavelength. However, this also means that DMI may not be suitable for analytes that require detection below 240 nm, as its background absorbance could interfere with sensitivity and baseline stability. The DMI:H_2_O mixture (cut-off at 240 nm) may be more appropriate for chromatographic methods operating at moderate UV wavelengths.

These findings are critical when evaluating DMI as a potential eluent in LC. While its moderate UV cut-off value is a limitation for low-wavelength detection, DMI remains a viable option for methods targeting analytes that absorb above 240 nm, especially in combination with water. Understanding these spectral characteristics is essential for optimizing LC method development, particularly in cases where the UV transparency of the eluent directly affects detection sensitivity and the signal-to-noise ratio.

### 2.5. Determination of the Partition Coefficient (logP) of DMI

**Figure 3:**
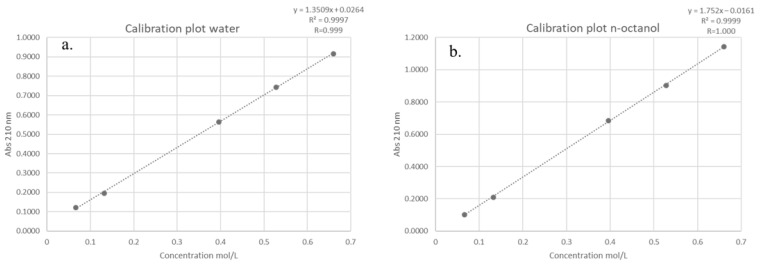
Calibration plot obtained during the determination of the *log P* value for: the (**a**) aqueous phaseand the (**b**) lipophilic phase (n-octanol).

The partition coefficient (*log P*) of DMI between n-octanol and water was determined according to the OECD Guideline 107 [33], which is applicable for compounds with *log P* values in the range of −2 to 4. A series of five standard solutions of DMI were prepared, and their absorbance values were measured at 210 nm in both the aqueous and lipophilic (n-octanol) phases using a UV-Vis spectrophotometer. The resulting absorbance-concentration data were used to construct calibration plots for each phase (Figure 3).

The calibration plots exhibited excellent linearity across the tested concentration range, with correlation coefficients (*R*) of 0.999 and 1.000 for the aqueous and lipophilic phases, respectively. Linear regression equations were derived for each phase, where absorbance (*y*) is expressed as a function of molar concentration (x, mol/L). Using these equations, absorbance values corresponding to a test concentration of 0.33 mol/L were calculated as 0.4722 for the aqueous phase and 0.5621 for the lipophilic phase.

Applying Beer–Lambert’s law, the molar absorptivity (*ε*) for each phase was determined by dividing the measured absorbance (*A*) by the product of molar concentration (*c*) and cuvette path length (*l*). The calculated ε values were 1.4309 L·mol^−1^·cm^−1^ for the aqueous phase and 1.7032 L·mol^−1^·cm^−1^ for the lipophilic phase.

After allowing the system to reach partition equilibrium, the absorbance of DMI in each phase was experimentally measured as 0.2635 (aqueous phase) and 0.1137 (lipophilic phase). These absorbance values were then converted into equilibrium concentrations, using the previously determined molar absorptivity. By dividing the absorbance (*A*) with the product of molar absorptivity (*ε*) and path length (*l*), the calculated equilibrium concentrations were 0.1841 mol/L for the aqueous phase and 0.0668 mol/L for the lipophilic phase. Finally, the partition coefficient was calculated as the logarithm of the ratio between the concentration of DMI in the lipid phase and its concentration in the aqueous phase. The obtained *log P* value of −0.44 indicates that DMI exhibits a greater affinity for the aqueous phase (water) than for the lipid phase (n-octanol).

This negative *log P* value suggests that DMI is more hydrophilic in nature. Although the magnitude of the value is not very large, it still reflects some degree of lipophilic character. However, overall, this solvent demonstrates a higher solubility in water compared to the lipid phase. Its hydrophilic nature and solubility in water suggest it could improve the separation efficiency and elution of polar compounds while ensuring compatibility with standard chromatographic systems.

### 2.6. Investigation of the Chromatographic Behavior of DMI Using Central Composite Face 2^3^ Experimental Design

Given the absence of literature data on the use of DMI as an eluent in LC, this study aimed to generate initial insights into its chromatographic behavior. In order to systematically explore the influence of relevant variables and to optimize experimental efficiency, a DoE strategy was employed. The chromatographic behavior of DMI was assessed on a C18 stationary phase using the Central Composite Face (CCF) 2^3^ experimental design. Toluene was selected as the model compound as it is commonly used as a retention marker for reversed-phase columns. The experimental design focused on assessing the effects of three independent variables as critical factors: the percentage (*v*/*v*) of DMI in the mobile phase, the percentage (*v*/*v*) of EtOH in the mobile phase, and the column temperature. Seventeen experiments were performed to evaluate the effect of the investigated factors on the viscosity of the mobile phase, the retention factor (*k*′) and the *P*_column_. The retention factor, *k*′, was calculated using the formula *k*′ = (*tR* − *t*0)/*t*0, where *tR* is the retention time of the analyte and *t*0 is the column dead time. Thiourea was used as a dead time marker (Table 5).

The percentage (*v*/*v*) of DMI in the mobile phase was investigated in the range of 25% to 45% (*v*/*v*), as lower concentrations may lead to insufficient elution strength, whereas higher concentrations could result in high viscosity, potentially impairing chromatographic performance.

EtOH was introduced as a factor both to enhance the separation capability of the mobile phase and to assess its impact on the viscosity of the mobile phase, with subsequent implications on *P*_column_. In addition to DMI and EtOH, H_2_O was used as the third component of the mobile phase. Its percentage (*v*/*v*) was inherently determined by the volumetric fractions of the other two eluents, resulting in an aqueous content ranging from 30% (*v*/*v*) to 70% (*v*/*v*). This range aligns with commonly used conditions in RP-HPLC and ensures compatibility with both the stationary phase and a broad range of analyte polarities. Preliminary findings indicated that temperature exerts a pronounced influence on DMI viscosity, necessitating a minimum column temperature of 40 °C to maintain acceptable flow mobile phase characteristics. The upper temperature limit was constrained to 60 °C to preserve the structural integrity of C18-bonded stationary phases, which are typically unstable beyond this range. In order to use DMI as an eluent in the mobile phase it should be pre-prepared as a solvent mixture, rather than system-based mixing within the HPLC. This is necessary because system-based mixing within the HPLC could introduce increased baseline noise due to the different viscosities of the solvents. Preparing the solvent mixture ensures better control over the solvent blend, minimizing viscosity fluctuations.

The primary responses monitored included: mobile phase viscosity, the *k*′ of toluene and *P*_column_, as these parameters are critical for the initial evaluation of the chromatographic behavior of DMI. Additionally, the peak symmetry factor (*As*) and the number of theoretical plates (*N*) were monitored throughout the experiments for informational purposes and were not included in the formal DoE model.

**Figure 4:**
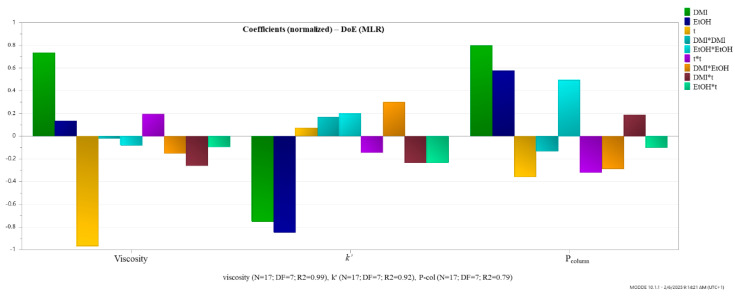
Regression coefficients plot of the influence of the experimental factors.

The obtained coefficient plots of the model (Figure 4) indicated that increasing the percentage (*v*/*v*) of DMI in the mobile phase increases both viscosity and *P*_column_, while increasing temperature has an inversely proportional effect. Regarding the influence of these two factors on the *k*′ of toluene, it can be seen that they have a negative effect, meaning that their increase leads to a reduced retention of the analyte. An increase in the percentage (*v*/*v*) of EtOH in the mobile phase has a negligible impact on viscosity, a negative effect on *k*′, and a positive effect on *P*_column_. This suggests that an increase in the percentage of EtOH in the mobile phase, along with DMI and H_2_O, is expected to reduce the analyte retention and increase the *P*_column_.

In addition to the linear terms, the model included quadratic (DMI*DMI, EtOH*EtOH, and t*t) and interaction terms (DMI*t, EtOH*t, and DMI*EtOH), revealing complex nonlinear and synergistic effects. The interpretations are as follows: DMI*DMI, represents the quadratic effect of DMI. It has a slight negative impact on viscosity, a mild positive effect on *k*′, and a negative effect on *P*_column_, indicating that higher concentrations of DMI may increase retention while slightly lowering viscosity and column pressure up to a certain point, beyond which these effects reverse. EtOH*EtOH, has a negative impact on viscosity, a positive effect on *k*′, and a notably positive effect on *P*_column_, suggesting that higher EtOH levels significantly raise backpressure while enhancing retention. The term t*t shows a positive influence on viscosity, and negative effects on both *k*′ and *P*_column_, suggesting a complex, non-linear relationship where temperature initially reduces retention and backpressure, but higher temperatures may lead to an increase in viscosity. The term DMI*EtOH, describes the interaction between DMI and EtOH. It has a negative effect on viscosity, a positive impact on *k*′, and a negative effect on *P*_column_, suggesting that this solvent combination can be tuned to reduce viscosity and column pressure while slightly improving retention. DMI*t presents the interaction between DMI and temperature. It negatively affects both viscosity and *k*′, but positively affects *P*_column_, indicating that the combined increase in DMI and temperature may lead to reduced retention and viscosity but increased backpressure. EtOH*t, shows the interaction between ethanol and temperature. It has mild negative effects across all responses, indicating a slightly detrimental synergistic impact when both factors are increased simultaneously. These interactions highlight the complex, often non-linear behavior of solvent mixtures and temperature in chromatographic systems. Understanding these coefficients is essential for optimizing solvent composition and operational temperature to achieve desirable performance metrics.

**Figure 5:**
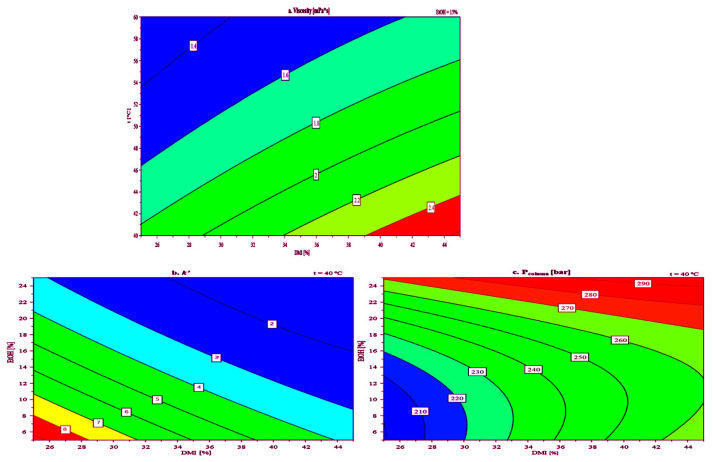
Response contour plot obtained with 2^3^ CCF DoE for: (**a**) viscosity, (**b**) retention factor (*k*′), and (**c**) column backpressure (*P*_column_).

After analyzing the regression factors, a response surface plot for viscosity (Figure 5a) as a function of the percentage (*v*/*v*) of DMI in the mobile phase and column temperature, at a constant value of EtOH percentage (15% *v*/*v*) was constructed. The EtOH percentage was chosen as a constant because it had the smallest influence on the viscosity of the mobile phase. Response surface plots for *k*′ and *P*_column_ as functions of the percentage (*v*/*v*) of DMI and EtOH in the mobile phase, at a temperature of 40 °C were also constructed (Figure 5b,c, respectively).

The viscosity diagram (Figure 5a) shows that increasing the DMI concentration in the mobile phase from 25% to 45% (*v*/*v*) generally leads to higher viscosity at both 40 °C and 60 °C. This trend aligns with the known behavior of DMI, where higher concentrations enhance intermolecular interactions, resulting in more viscous solutions. As expected, viscosity values were lower at 60 °C compared to 40 °C for the same solvent compositions, indicating reduced intermolecular forces at elevated temperatures. Notably, even at 40 °C, the mobile phase containing 45% (*v*/*v*) DMI maintained an acceptable viscosity of 2.4 mPa·s. The *P*_column_ diagram plot (Figure 5c) indicates that at 40 °C, using a mobile phase with 45% (*v*/*v*) DMI and 25% (*v*/*v*) EtOH results in an acceptable *P*_column_ of approximately 300 bar. This suggests that while increased mobile phase viscosity does raise flow resistance, appropriate temperature and solvent composition can keep *P*_column_ within operational limits, which is critical for stable chromatographic performance. A lower value for the *P*_column_ of around 200 bar was observed when using a mobile phase consisting of 25% (*v*/*v*) DMI, 5% (*v*/*v*) EtOH, and 70% (*v*/*v*) H_2_O at 40 °C. The *k*′, used to evaluate analyte retention and solvent-stationary phase interaction, was analyzed via response surface plots (Figure 5b). Higher DMI concentrations in the mobile phase led to lower *k*′ values for toluene, confirming DMI’s stronger elution strength compared to water. The *k*′ values for toluene ranged from 2 to 8 as DMI concentration decreased from 45% to 25% (*v*/*v*). In all experiments, the *As* of toluene remained within the acceptable limits (<1.8) for peak symmetry, ranging from 1.1 to 1.3, while the *N* ranged from 1378 to 7485. This further confirms that DMI can be effectively used as an eluent in LC analyses.

This study provides initial insights into the chromatographic behavior of DMI as an eluent in LC. Despite its inherently higher viscosity compared to conventional organic solvents, DMI was successfully employed in the mobile phase at concentrations up to 45% (*v*/*v*) without exceeding acceptable operational *P*_column_, provided the system temperature was maintained at or above 40 °C. Under these conditions, a *P*_column_ below 300 bar was achieved, indicating that the higher viscosity of DMI can be effectively managed through thermal control.

The use of EtOH as a co-eluent further enhanced the flexibility of the mobile phase composition, enabling improved selectivity in separations where higher resolution is required. The performance of DMI as an eluent was found to be comparable to that of widely used solvents such as ACN and MeOH. Namely, DMI demonstrated a higher elution strength relative to H_2_O, as reflected by the observed reduction in analyte retention time with increasing DMI content in the mobile phase.

### 2.7. Investigation of the Applicability of DMI as an Eluent in Chromatographic Analyses

To confirm the applicability of DMI as an eluent in chromatographic analyses, a HPLC method was developed for the determination of methylparahydroxybenzoate (MPHB) in Carbocysteine oral solution and the determination of MPHB and propylparahydroxybenzoate (PPHB) in Loratadine oral solution and Paracetamol oral solution. Conventional RP-HPLC methods for the quantification of MPHB and PPHB typically use MeOH as an organic eluent in the mobile phase, with a column temperature of 25 °C [34,35,36]. In this study, MeOH from the conventional method (described in Section 3.8) was replaced with DMI, resulting in a mobile phase composed of 40% DMI and 60% H_2_O for the analysis of preservatives in the selected formulations. Due to the higher viscosity of DMI, the column temperature was increased from 25 °C to 50 °C. These adjustments to the mobile phase composition and temperature were the only modifications introduced; all other chromatographic conditions remained consistent with the conventional method.

This modification led to the development of a generic method for preservative determination in oral solutions, providing enhanced separation efficiency. Given that this is the first application of DMI as an eluent in chromatographic analyses, particular attention was paid to evaluating key chromatographic parameters: retention time (*Rt*), *As*, *k*′, *N*, and *P*_column_. These parameters were compared to those obtained using the conventional method.

**Figure 6:**
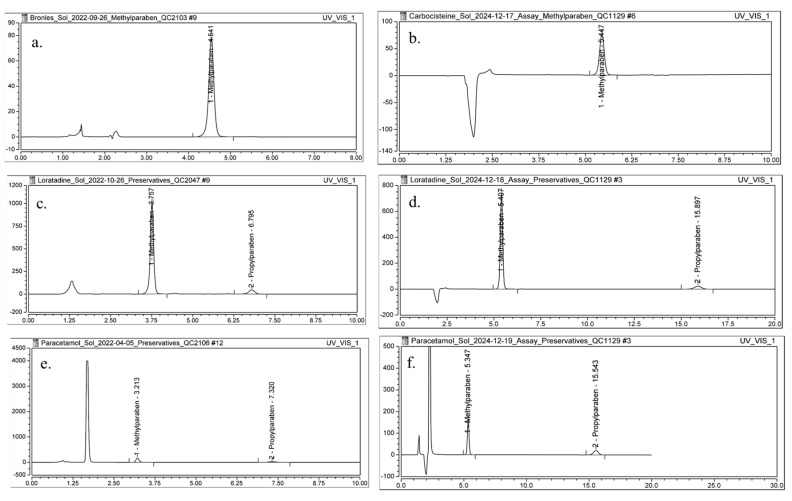
Chromatograms of a sample solution for: (**a**) Carbocysteine oral solution 250 mg/5 mL, conventional method, (**b**) Carbocysteine oral solution 250 mg/5 mL, method with DMI, (**c**) Loratadine oral solution 1 mg/mL, conventional method, (**d**) Loratadine oral solution 1 mg/mL, method with DMI, (**e**) Paracetamol oral solution 120 mg/5 mL, conventional method, and (**f**) Paracetamol 120 mg/5 mL oral solution, method with DMI.

The *Rt* of MPHB for the Carbocysteine oral solution obtained with the conventional method (Figure 6a) increased from 4.54 min to 5.45 min obtained with method with DMI (Figure 6b). This increase can be attributed to the elevated column temperature and the altered mobile phase composition, which likely enhanced interactions between the analyte and the stationary phase. A slight improvement in peak *As* was observed, with *As* increasing from 0.95 to 0.97. The *N* increased from 6834 to 8561, indicating enhanced column efficiency under the optimized mobile phase conditions. The *k*′ also increased from 2.78 to 3.19, suggesting greater retention of MPHB on the stationary phase. Additionally, *P*_column_ was reduced from 234 bar to 191 bar (Table 6).

In the analysis of parabens in the Loratadine oral solution, the method utilizing DMI in the mobile phase (Figure 6d) also demonstrated an increase in both *Rt* and *k*′ for MPHB and for PPHB when compared to the conventional method (Figure 6c). Additionally, similar to the previous case, improvements were observed in peak *As* and column efficiency. The *P*_column_ slightly decreased from 198 bar to 192 bar, indicating favorable adjustments in flow dynamics (Table 6).

The determination of MPHB and PPHB content in the Paracetamol oral solution using DMI as mobile phase constituent (Figure 6f) resulted in improved and satisfactory values for the evaluated chromatographic parameters compared to the conventional method (Figure 6e, Table 6).

The developed method employing DMI as an organic eluent in the mobile phase enabled higher values of the *k*′ values of the analytes, improved peak *As* for both MPHB and PPHB, and enhanced column efficiency. Additionally, the optimized method, which utilizes a mobile phase composed of H_2_O and DMI along with an elevated column temperature, resulted in lower *P*_column_, thereby contributing to the overall efficiency of the chromatographic process. The analysis time for the determination of both preservatives using the developed method was extended to 20 min, compared to 10 min with the conventional method. Although the analysis time is extended, the use of this greener solvent system contributes to decreased chemical toxicity, improved operator safety, and better compliance with the principles of GAC. Therefore, the increased run time is justified by the method’s improved environmental compatibility and its potential for long-term application in sustainable pharmaceutical analysis.

During this study satisfactory short- to medium-term compatibility of DMI with reversed-phase C18 silica-based columns was observed, as evidenced by the absence of column degradation or deterioration in chromatographic performance—including stable peak shapes, retention times, and column efficiency—even after numerous injections (over 130 combined across different column types and lengths). These findings suggest that DMI can be reliably employed under the tested conditions without compromising column integrity or analytical quality. However, this study was limited to a relatively narrow range of stationary phases and chromatographic conditions. In particular, the long-term stability of DMI and its potential interactions with other bonded phase chemistries (such as phenyl, cyano, and bare silica columns) remain unexplored. Additionally, all experiments were conducted using a single batch of chromatographic columns, which may not fully capture the variability inherent in column manufacturing. Therefore, further comprehensive studies assessing the long-term effects of DMI under extended use, as well as its compatibility across diverse column chemistries and multiple column batches, are crucial to fully establish its suitability for routine chromatographic applications.

## 3. Materials and Methods

### 3.1. Model Compounds and Reagents

Dimethyl isosorbide (BioRenewable, ReagentPlus^®^, ≥99%) was obtained from Sigma Aldrich (St. Louis, MO, USA), while ethanol gradient grade for liquid chromatography LiChrosolv^®^, was procured from Merck KGaA Darmstadt Germany. The analytical standards used in this study included toluene for analysis EMSURE^®^, ACS, ISO, Reag. Ph Eur obtained from Merck KGaA Darmstadt Germany, which served as a model compound to evaluate the chromatographic behavior of DMI. Additionally, methylparaben (MPHB) and propylparaben (PPHB) were used as in-house secondary reference standards (RS), both obtained from Alkaloid AD Skopje. Pharmaceutical formulations analyzed in the study included Carbocysteine oral solution (250 mg/5 mL), Loratadine oral solution (1 mg/mL), and Paracetamol oral solution (120 mg/5 mL), all of which were supplied by Alkaloid AD Skopje (Skopje, North Macedonia).

### 3.2. Experimental Setup and Equipment Used

For the experimental procedures, viscosity measurements were performed using an Anton Paar MCR 92 Viscometer (Anton Paar GmbH, Graz, Austria). UV cut-off determination was conducted using a UV-visible spectrophotometer (UV-2600) from Shimadzu (Kyoto, Japan), with a wavelength range of 185–900 nm. The partition coefficient was determined using a UV-Vis Spectrophotometer (8453, Agilent Technologies GmbH, Waldbronn, Germany). Chromatographic analyses were carried out on an Agilent 1260 Infinity Quaternary LC with UV-vis detector (Agilent Technologies GmbH, Waldbronn, Germany), particularly for the Design of Experiments (DoE) studies. The investigation of the applicability of DMI as an eluent for the determination of parabens in oral solutions was performed on a Dionex UltiMate 3000 (UHPLC) Thermo Scientific with a DA detector (Thermo Scientific, Sunnyvale, CA, USA). A regenerated cellulose (RC) membrane filter (Sartorius, Göttingen, Germany) of 0.45 µm was used for the filtration of the solutions.

### 3.3. Selection Methodology

The selection and evaluation of DMI was performed based on several key parameters according to the CHEM21 criteria and Safety Data Sheets (SDS) [11,12,24]. These parameters include: solvent classification, boiling point, incineration, recycle, biotreatment, VOC emissions, environmental impact (aquatic and air), health hazard, exposure potential, flammability and explosion risk, sensitization, reactivity. These parameters were used to assess the overall suitability of DMI as a solvent in terms of environmental, health, safety, and chemical properties.

### 3.4. Miscibility Experiments

The method for determining the miscibility of DMI with H_2_O involves the preparation of mixtures with varying ratios (*v*/*v*) of DMI to H_2_O (10:90, 20:80, 30:70, 40:60 and 50:50, and vice versa). These mixtures were prepared and tested for miscibility at room temperature. The miscibility wass evaluated by observing whether the mixture formed a homogeneous phase or if phase separation occurred, indicating immiscibility.

### 3.5. Viscosity Measurements

The measurements were carried out at 25 °C, 40 °C, and 60 °C for pure DMI and its mixtures with H_2_O and EtOH. The following DMI:H_2_O ratios (*v*/*v*) were tested: (70:30, 50:50 and 30:70). For each sample, the viscosity was measured at different temperatures (25 °C, 40 °C, and 60 °C). Additionally, mixtures of DMI, H_2_O and EtOH in various ratios (*v*/*v*) (33.33:33.33:33.33, 50:25:25, 25:50:25, and 25:25:50) were also analyzed for their viscosities at 40 °C and 60 °C.

### 3.6. UV Cut-Off Determination

The UV cut-off of pure DMI and its mixture with H_2_O (50:50 (*v*/*v*)) was determined using a UV-visible spectrophotometer. The process for determining the UV cut-off was as follows: pure DMI and a mixture of DMI and H_2_O 50:50 (*v*/*v*) were prepared. The samples were scanned over the wavelength range of 200–400 nm using the UV-visible spectrophotometer. The absorption spectrum of each sample was recorded, noting the wavelengths at which significant absorbance occurred (1.0 AU).

### 3.7. Partition Coefficient Determination

The *logP* was determined using the OECD 107—partition coefficient (n-octanol/water): shake flask method [33]. This method involves equilibrating duplicate samples of H_2_O, n-octanol, and the test compound via mechanical shaking. The two phases (aqueous and octanol) were separated by centrifugation, and the concentrations of the test compound in each phase were determined using the UV/vis spectrophotometric method at a wavelength of 210 nm.

### 3.8. Design of Experiments (DoE) for Investigation of the Chromatographic Behavior of DMI

DoE was applied for the investigation of the chromatographic behavior of DMI. The experimental design was developed using the CCF 2^3^ design using the MODDE 10.1.1 software (Umea, Sweden). The 2^3^ CCF DoE required 17 experiments (2k + 2k + n experiments, where k was the number of parameters studied and n was the number of central points included, n = 3). The Chromatographic behavior of DMI was investigated through three factors: the content of DMI in the mobile phase (25% to 45% (*v*/*v*)), the content of EtOH in the mobile phase (5% to 25% (*v*/*v*)) and the column temperature (40 °C to 60 °C), as shown in Table 4. Experimental conditions, including solvent composition and temperature, were specifically adjusted to achieve various levels of viscosity and *P*_column_. The evaluated responses were the viscosity of the mobile phase (mPa∙s), the *P*_column_ in bar, and the *k*′ of toluene. Chromatographic separations for the DoE experiments were conducted on a C18 stationary phase (LiChrospher^®^ 100RP-18 (5 μm) Hibar^®^ RT 125-4 HPLC column). The mobile phase flow rate was maintained at 1 mL/min for all experiments, with an injection volume of 5 µL and the detection wavelength for toluene was set at 254 nm.

### 3.9. Conventional Method and Method Using DMI as an Eluent in the LC Mobile Phase for Determining Preservative Content in Carbocysteine, Loratadine, and Paracetamol Oral Solutions

The conventional HPLC method, validated in accordance with the ICH Q2 guideline [37], was used for the determination of parabens in oral solutions. The chromatography was performed on a LiChrospher 100 RP 18, 250 mm × 4 mm i.d., 5 µm. The mobile phase consisted of MeOH and H_2_O in a 60:40 (*v*/*v*) ratio, which was filtered through a 0.45 µm RC membrane filter. The flow rate was set to 1.0 mL/min, the injection volume was 20 μL and the column temperature was maintained at 25 °C. Detection was performed at a wavelength of 254 nm.

The chromatographic conditions were the same as for the method with MeOH, except for the composition of the mobile phase and the column temperature. The optimized method used a mobile phase consisting of DMI and H_2_O in a 40:60 (*v*/*v*) ratio, whereas the column temperature was 50 °C.

### 3.10. Preparation of Standard Solutions and Test Solutions for the Determination of MPHB and PPHB in Oral Solutions

#### 3.10.1. Carbocisteine Oral Solution 250 mg/5 mL

The standard solution was prepared by dissolving 150 mg of MPHB RS in 10 mL of DMI. The dilution to the final concentration of 0.006 mg/mL was made with purified water. The sample solution was prepared by transferring 5.0 mL of the test solution (Carbocysteine oral solution 250 mg/5 mL) into a flask, adding 1.0 mL of DMI, and diluting to the final concentration of 0.006 mg/mL with purified water.

#### 3.10.2. Paracetamol Oral Solution 120 mg/5 mL

The stock standard solution 1 was prepared by dissolving approximately 28 mg of MPHB RS in a 100.0 mL volumetric flask, using a solvent composed of DMI and H_2_O in a 40:60 *v*/*v* ratio. The stock standard solution 2 was prepared by dissolving approximately 12 mg of PPHB RS in a 100.0 mL volumetric flask, using the same solvent (DMI:H_2_O = 40:60 (*v*/*v*)). Standard solution 3 was prepared by mixing 5.0 mL of standard solution 1 and 5.0 mL of standard solution 2 in a 100.0 mL volumetric flask, followed by dilution to the final volume with purified water to obtain the combined standard solution for the analysis of Paracetamol oral solution (final concentrations: MPHB 0.014 mg/mL and PPHB 0.006 mg/mL).

The sample solution was prepared by transferring 1.0 mL of the test solution (Paracetamol oral solution 120 mg/5 mL) into a 50.0 mL volumetric flask, adding 5.0 mL of DMI, and filling to the final volume with purified water (final concentrations: MPHB 0.014 mg/mLand PPHB 0.006 mg/mL).

#### 3.10.3. Loratadine Oral Solution 1 mg/mL

The stock standard solution 1 was prepared by weighing approximately 75 mg of MPHB RS into a 50.0 mL volumetric flask. The substance was dissolved and diluted to the final volume with a solvent composed of DMI and H_2_O in a 40:60 (*v*/*v*) ratio. The stock standard solution 2 was prepared by weighing approximately 30 mg of PPHB RS into a 200.0 mL volumetric flask. The substance was dissolved and diluted to the final volume with the same solvent (DMI:H_2_O = 40:60 (*v*/*v*)). Standard solution 3 was prepared by transferring 2.0 mL of standard solution 1 and 2.0 mL of standard solution 2 into a 50.0 mL volumetric flask, followed by dilution to the final volume with purified water (final concentrations: MPHB 0.06 mg/mL and PPHB 0.006 mg/mL).

The sample solution was prepared by transferring 3.0 mL of the test solution (Loratadine oral solution 1 mg/mL) into a 50.0 mL volumetric flask, adding 1.6 mL of DMI, and filling to the final volume with purified water (final concentrations: MPHB 0.06 mg/mL and PPHB 0.006 mg/mL).

## 4. Conclusions

In this study, the applicability of the bio-renewable solvent DMI as an eluent in chromatographic analyses was investigated for the first time. Its favorable characteristics, including a beneficial environmental and health profile and water solubility make it an ideal candidate for use as an organic eluent in the mobile phase in LC.

The experimentally determined value for the partition coefficient of DMI (*logP* = −0.44) reflects its hydrophilic nature and supports its suitability in chromatographic systems for the separation of polar analytes. In addition, DMI is miscible with water in all tested ratios, further confirming its versatility and reliability as an eluent in chromatographic analyses. The UV cut-off value for a DMI:H_2_O mixture, 50:50 (*v*/*v*) was found to be 240 nm, making it suitable for chromatographic analyte detection within the UV region of the electromagnetic spectrum.

The chromatographic behavior of DMI, as evaluated using a 2^3^ CCF experimental design, indicates that a mobile phase containing up to 50% (*v*/*v*) DMI can be effectively utilized at a column temperature of 40 °C, resulting in an operating pressure of approximately 300 bar. These results suggest that DMI exhibits acceptable viscosity for chromatographic use at concentrations up to 50% (*v*/*v*), provided that appropriate temperature conditions are maintained. Specifically, a minimum column temperature of 40 °C is recommended to ensure manageable viscosity and *P*_column_. Given the inherently high viscosity of pure DMI (6.70 mPa·s at 25 °C) it should not be introduced into the HPLC system in its neat form; rather, it must be incorporated as a pre-prepared mixture with H_2_O and/or EtOH to ensure proper solvent handling and system compatibility.

The applicability of DMI as an eluent in chromatographic analyses was confirmed through its implementation as an eluent in a RP-HPLC method for determining the content of MPHB and PPHB in three oral solution formulations. The results confirmed that replacing MeOH with DMI in the mobile phase, in combination with elevated column temperature, led to improved peak *As*, increased column efficiency, and reduced *P*_column_.

The investigations demonstrated satisfactory short- to medium-term compatibility of DMI with reversed-phase C18 silica-based columns. However, the current study did not cover the long-term impact of solvent mixtures on column longevity and separation efficiency. Future systematic evaluations are recommended to ensure sustained analytical performance over extended use. Mathematical modeling of temperature-dependent viscosity, remains a promising avenue for future work to quantitatively predict DMI’s chromatographic behavior under varying thermal conditions.

Overall, DMI represents a promising bio-renewable solvent that offers significant potential as a substitute for conventional organic solvents in chromatographic analyses, contributing to both environmental sustainability and improved chromatographic performance. However, its application in LC is viable only under well-defined experimental conditions. The continued optimization of conventional chromatographic methods throughout their lifecycle will further underscore the relevance of DMI in various analytical contexts, particularly within the framework of green chemistry and sustainable analytical practices.

## Figures and Tables

**Table 1:** Comparison of DMI versus selected green and non-green solvents based on green solvent criteria—extracted from the GSK Solvent Sustainability Guide [12].

Parameter	DMI	DMC	Cyrene	MeOH	EtOH	Rating Scale
VOC Emissions	10	5	10	3	4	1 = very high, 10 = very low
Aquatic Impact	9	9	9	10	9	1 = very high, 10 = very low
Air Impact	6	7	6	7	5	1 = very high, 10 = very low
Health Hazard	4	10	4	4	10	1 = very high, 10 = very low
Exposure Potential	9	6	8	6	8	1 = high, 10 = very low
Flammability/Explosion Risk	9	6	10	5	6	1 = very high, 10 = very safe
Reactivity	8	10	10	10	10	1 = highly reactive, 10 = stable
Biotreatability	5	5	5	3	3	1 = poor, 10 = excellent
Recycle Potential	4	3	4	4	5	1 = very low, 10 = high
Incineration Suitability	4	4	4	4	5	1 = poor, 10 = excellent

Legend: 1–3 (red): unfavorable effect/high risk/low suitability, 4–6 (yellow): moderate effect/moderate risk/acceptable suitability, 7–8 (light green): good effect/low risk/good suitability, 9–10 (dark green): very favorable effect/very low risk/excellent suitability.

**Table 3:** Results from viscosity measurements of mixtures containing different ratios of DMI and H_2_O at different temperatures.

Sample	Viscosity (mPa·s) at 25 °C (Mean ± SD, n = 3)	Viscosity (mPa·s) at 40 °C (Mean ± SD, n = 3)	Viscosity (mPa·s) at 60 °C (Mean ± SD, n = 3)
Pure DMI	6.70 ± 0.10	4.53 ± 0.32	2.79 ± 0.03
70% DMI:30% H_2_O	6.38 ± 0.08	3.84 ± 0.01	2.40 ± 0.23
50% DMI:50% H_2_O	3.99 ± 0.11	2.73 ± 0.11	1.64 ± 0.01
30% DMI:70% H_2_O	2.38 ± 0.01	1.68 ± 0.01	1.08 ± 0.02

**Table 4:** Results from viscosity measurements of mixtures containing different ratios of DMI, H_2_O and EtOH at different temperatures.

Sample	Viscosity (mPa·s) at 25 °C (Mean ± SD, n = 3)	Viscosity (mPa·s) at 40 °C (Mean ± SD, n = 3)	Viscosity (mPa·s) at 60 °C (Mean ± SD, n = 3)
33.33% DMI:33.33% H_2_O: 33.33% EtOH	2.31 ± 0.02	2.29 ± 0.02	1.49 ± 0.02
50% DMI:25% H_2_O: 25% EtOH	2.51 ± 0.05	2.62 ± 0.10	1.76 ± 0.01
25% DMI:50% H_2_O: 25% EtOH	1.88 ± 0.02	1.97 ± 0.08	1.29 ± 0.03
25% DMI:25% H_2_O: 50% EtOH	2.01 ± 0.02	2.01 ± 0.01	1.28 ± 0.01

**Table 5:** Critical factors and obtained responses for the investigation of the chromatographic behavior of DMI using the CCF 2^3^ design.

Exp. Name	Experimental Factors			Responses		
	DMI % (*v*/*v*)	EtOH % (*v*/*v*)	Temperature (°C)	Viscosity (mPa·s)	*k*′	*P*_column_ (Bar)
N1	25	5	40	1.629	7.6	200
N2	45	5	40	2.632	4.6	286
N3	25	25	40	1.98	3.9	277
N4	45	25	40	2.564	0.7	304
N5	25	5	60	1.242	13.7	164
N6	45	5	60	1.656	3.4	262
N7	25	25	60	1.264	2.7	210
N8	45	25	60	1.621	0.6	281
N9	25	15	50	1.509	8.1	217
N10	45	15	50	2.027	1.6	233
N11	35	5	50	1.627	8.4	221
N12	35	25	50	1.862	1.5	276
N13	35	15	40	2.241	4.4	210
N14	35	15	60	1.465	3.2	226
N15	35	15	50	1.805	3.6	261
N16	35	15	50	1.797	3.6	261
N17	35	15	50	1.786	3.6	260

**Table 6:** Comparison of chromatographic performance between conventional and optimized methods with DMI for Carbocysteineoral solution 250 mg/5 mL, Loratadine oral solution 1 mg/mL and for Paracetamol oral solution 120 mg/5 mL.

Chromatographic Parameter	CarbocysteineOral Solution 250 mg/5 mL		Loratadine Oral Solution 1 mg/mL		Paracetamol Oral Solution 120 mg/5 mL	
	Conventional Method *	Method with DMI **	Conventional Method *	Method with DMI **	Conventional Method *	Method with DMI **
*Rt* MPHB (min)	4.54	5.45	3.76	5.41	3.21	5.35
*As* MPHB (EP)	0.95	0.97	0.95	0.99	1.12	0.99
*N* MPHB (EP)	6834	8561	6834	8467	5256	7704
*k*′ MPHB	2.78	3.19	1.89	3.16	2.21	3.11
*Rt* PPHB (min)	/	/	6.80	15.90	7.32	15.54
*As* PPHB (EP)	/	/	0.90	0.95	1.10	0.96
*N* PPHB (EP)	/	/	6591	8202	5276	7511
*k*′ PPHB	/	/	4.23	11.23	6.30	10.96
*P*_column_ (bar)	234	191	198	192	356	198

Legend: Conventional method *—mobile phase water and methanol (40:60 *v*/*v*%) and, column temperature 25 °C; optimized method with DMI **—mobile phase water and DMI (60:40 *v*/*v*%) and column temperature 50 °C.

## Data Availability

Data presented in this study are available upon request.

## Abbreviations

ACN	Acetonitrile
ACS	American Chemical Society
*As*	Asymmetry Factor (Peak Symmetry)
C18	Octadecylsilane-bonded Stationary Phase
CCF	Central Composite Face
CHEM21	Chemical 21st Century (Sustainable Chemistry Initiative)
°C	Degrees Celsius
DMI	Dimethyl Isosorbide
DoE	Design of Experiments
EP/Ph Eur	European Pharmacopoeia
est	Estimated
EtOH	Ethanol
GAC	Green Analytical Chemistry
GSK	GlaxoSmithKline
H_2_O	Water
HPLC	High-performance Liquid Chromatography
ISO	International Organization for Standardization
*k*′	Retention Factor (Capacity Factor)
L/mol·cm	Liters per Mole per Centimeter (Unit for Molar Absorption Coefficient)
LC	Liquid Chromatography
*log P*	Partition Coefficient (Logarithmic Scale)
MeOH	Methanol
mol/L	Moles per Liter (Concentration Unit)
MPHB	Methylparahydroxybenzoate/Methylparaben
mPa·s	Millipascal Second (Unit of Viscosity)
*N*	Number of Theoretical Plates
nm	Nanometers (Wavelength Unit)
OECD	Organisation for Economic Co-operation and Development
*P*_column_	Column Backpressure
PPHB	Propylparahydroxybenzoate/Propylparaben
RC	Regenerated Cellulose
RP-HPLC	Reverse-Phase High-Performance Liquid Chromatography
*R*	Correlation Coefficient
RS	Reference Standard
*Rt*	Retention Time
SDS	Safety Data Sheet
UV	Ultraviolet
UHPLC	Ultra-High-Performance Liquid Chromatography
*v*/*v*	Volume/Volume ratio
VOC	Volatile Organic Compound
